# The Effectiveness of a Cover-Copy-Compare App for Developing Secondary School Students’ Mathematical Procedural Fluency with Straight-Line Graphs

**DOI:** 10.1007/s42330-025-00381-1

**Published:** 2025-09-05

**Authors:** Jacob Strauss, Colin Foster, Tim Jay

**Affiliations:** 1 Department of Mathematics Education, Loughborough University, Schofield Building, Loughborough, LE11 3TU UK; 2 School of Education, University of Nottingham, Dearing Building, Jubilee Campus, Wollaton Road, Nottingham, NG8 1BB UK

**Keywords:** Cover-copy-compare, Secondary mathematics education, Software application, Procedural fluency, Straight-line graphs

## Abstract

Cover-copy-compare (CCC) is a simple, self-managed process that enables students to develop fluency in important facts and procedures through modelling, practice and corrective feedback. CCC has been studied at primary-school-level mathematics, but not with the complex, multi-step kinds of procedures used at secondary school (age 11 and above). This paper reports on a randomised controlled trial involving 224 students (ages 14–16) comparing a novel CCC app to a non-CCC app. Both apps used similar technological environments to teach students how to find the equation of a straight line given two points. We found that students using the CCC app improved their procedural fluency more than those completing standard exercises did. We conclude that CCC has the potential to be used in secondary education and offer suggestions for future research.

## Résumé

L’approche « recouvrir-recopier-comparer» (RRC) est un procédé simple et autogéré qui permet aux élèves d’acquérir une maîtrise des faits et procédures importants grâce à la modélisation, la pratique et la rétroaction corrective. Le RRC a été étudié dans le cadre des mathématiques enseignées à l’école primaire, mais jamais avec les procédures complexes en plusieurs étapes qui sont utilisées au secondaire (à partir de 11 ans). Cet article rend compte d’un essai clinique randomisé mené auprès de 224 élèves (âgés de 14 à 16 ans) comparant une nouvelle application RRC à une application n’utilisant pas le RRC. Les deux applications employaient des environnements technologiques similaires pour enseigner aux élèves comment déduire l’équation d’une droite à partir de deux points. Nous avons constaté que les élèves utilisant l’application RRC ont davantage amélioré leur maîtrise des procédures que ceux qui ont effectué des exercices traditionnels. Nous concluons que le RRC peut être utilisé dans le cadre de l’enseignement au secondaire et proposons des pistes pour de futures recherches.

## Introduction

Enhancing students’ procedural fluency, defined as the ability to apply mathematical procedures accurately, efficiently and flexibly (National Council of Teachers of Mathematics, 2014), is an important objective for secondary school mathematics teaching. Students’ capacity to accurately and efficiently solve routine questions is a significant predictor of their achievement on standardised mathematics tests (Awofala, 2017; Samuelsson, 2011). Procedural fluency is recognised as important to students’ ability to reason mathematically and solve problems, which are the ultimate goals of learning mathematics and learning to be a mathematician (Foster, 2018; McNeil et al., 2025).

Neurological research has identified that, while answering single-digit arithmetic questions, secondary school students with higher mathematics grades engaged brain mechanisms associated with fact retrieval (left supramarginal gyrus and bilateral anterior cingulate cortex), whereas students with lower grades activated quantity processing mechanisms (right intraparietal sulcus) (Price et al., 2013). These findings point to the importance of procedural fluency: automaticity with mathematical facts frees up cognitive resources to attend to more complex problems (Clarke et al., 2016; Foster, 2018; Sweller, 2011). Automatisation is especially important for secondary students, as they are required to solve complex, multi-step problems and ‘move fluently between representations of mathematical ideas’ (DfE, 2014, p. 160). While an excessive focus on developing procedural fluency impoverishes students’ experiences of mathematics (Liljedahl, 2020), some focus on fluency within a balanced approach helps to equip students to tackle stimulating mathematical tasks (Foster, 2018).

One approach to developing students’ mathematical fluency is cover-copy-compare (CCC), which is a simple, self-managed learning method that incorporates three components: modelling, practice and corrective feedback (Anghileri, 2006; Skinner et al., 1989; Riccomini et al., 2017). (Here, by ‘modelling’ we do not mean ‘mathematical modelling’ but demonstrating a procedure to students that they are then meant to imitate.) In its paper-based form, students are given a worksheet of questions and a separate sheet of model solutions to the same questions. For each question, students look at the model solution; then, they cover it with a piece of paper (‘Cover’) and attempt to answer the question by themselves (‘Copy’). Once students have attempted the question, they uncover the model solution and ‘Compare’ it with what they have done (Skinner et al., 1989). Reviews and meta-analyses have shown CCC to be effective in helping students improve their mathematics performance (Codding et al., 2011; Joseph et al., 2012; Stocker & Kubina, 2017). However, the vast majority of studies have been conducted with primary-age students, and the few that have been done with secondary-age students have involved students with special educational needs (Codding et al., 2011; Joseph et al., 2012).

This paper reports the first empirical study to evaluate the use of CCC in secondary education focusing on mathematical content, and the first to develop a computerised implementation of CCC. We chose the content area of straight-line graphs and the specific procedure of finding the equation of a straight line given the pairs of coordinates of two points on the line. Evidence from a randomised controlled trial addresses an important gap in the literature and has implications for researchers and practitioners on how to design pedagogical approaches to develop secondary school students’ procedural fluency.

## Cover-Copy-Compare

Alptekin and Sönmez (2022) published a systematic review of intervention studies that investigated CCC as a method to improve students’ mathematical fluency. They reviewed 22 studies, analysing the methodological approaches, characteristics of the participants, educational setting, effectiveness, maintenance and generalisation of the learned skill and the participants’ perception of CCC. They found that CCC was most often used to develop fluency with basic arithmetic in students aged 7–12. Twenty of the studies in their review were characterised as having a single-subject design, and two of them were quasi-experimental. All of the studies except one (Schrauben & Dean, 2019) showed an improvement in participants’ fluency with basic mathematics facts. Alptekin and Sönmez (2022) did not provide a metric for the overall effectiveness of CCC, and they highlighted a need for more experimental studies that investigate the efficacy of CCC in other settings and with other participants.

### Components of CCC

Riccomini et al., (2017) offered a theoretical explanation of the repeatedly observed benefits of CCC. They argued that effective fluency practice should incorporate three key elements: (i) modelling, (ii) multiple opportunities to respond, and (iii) immediate feedback. Riccomini et al., (2017) argued that CCC is effective because these three elements are embedded in its design. Each of these elements has been studied individually, and in this section we review the literature on all three.

(i) Modelling

Modelling is defined as presenting an idealised solution that a learner is meant to imitate (Anghileri, 2006).^1^ The model solution comprises a series of concise steps leading to the answer to the question, alongside a written comment describing how each step fits into the wider solution. In their meta-analysis of 17 basic-fact fluency interventions, Codding et al. (2011) found that interventions incorporating some form of modelling produced the largest treatment effect sizes (mean *φ* of 0.71). This notion of modelling is closely related to studies on worked examples in the mathematics education literature (e.g. Renkl, 2017), where students’ performance is improved by being first presented with worked-out examples that are similar to the questions that they are going to go on to attempt.

(ii) Multiple Opportunities to respond

The concept of ‘opportunities to respond’ (OTR) refers to the number of times that a learner is given the opportunity to correctly respond to a question, task or demand. There is little literature specifically investigating the effects of OTR on learning outcomes. However, in the specific case of CCC, Codding et al. (2019) compared the spacing of practice sessions with the number of OTR by giving different experimental groups different numbers of CCC questions. In a pre-, post- and delayed post-test intervention study, Codding et al. (2019) randomly assigned 112 second- and third-grade (ages 7–9) students to one of four conditions: (i) distributed practice, low OTR; (ii) massed practice, low OTR; (iii) distributed practice, high OTR; and (iv) massed practice, high OTR. Here, distributed (or ‘spaced’) practice is practice that takes place over time with gaps in between, in contrast to massed practice, which takes place altogether at one time. Codding et al. operationalised low and high OTR as answering either 30 or 60 CCC arithmetic questions. After controlling for grade-level mathematics knowledge in a multilevel prediction model, they found that OTR — but not practice spacing — was a significant predictor of students’ test scores a week after the intervention was conducted.

(iii) Immediate Feedback

Feedback is information provided to a student regarding an aspect of their performance or understanding (Hattie & Timperley, 2007). Feedback has been shown to be important to developing students’ mathematical fluency (Duhon et al., 2015) by providing invaluable formative assessment (Burkhardt & Schoenfeld, 2019; Wiliam, 2006). As part of a randomised controlled trial on the effects of immediate feedback on students’ mathematical fluency, Duhon et al. (2015) randomly divided 48 second-grade students into one of three groups: (i) a control group that did nothing between the pre- and post-test, (ii) a group that completed a computerised ‘Explicit Timing’^2^ intervention without feedback, and (iii) a group that completed the same intervention *with* feedback. A repeated-measures ANOVA found that the ‘with feedback’ group had a significantly higher mean improvement than the ‘without feedback’ group (Glass’s *Δ* = 3.52), which had a significantly higher mean improvement than the control group (Glass’s *Δ* = 2.44).

### CCC in Secondary Education

Only a few studies have addressed the use of CCC in secondary school (age 11–18) in any subject. In an interview study with 20 high-school students, Althobaiti (2020) found that students believed that a spelling instruction CCC was effective and enjoyable. Morano and Aigotti (2022) proposed how CCC could be used to develop students’ procedural fluency with compound area calculations, and offered suggestions on how to implement a CCC activity in secondary mathematics classrooms. Finally, Inouye (2018) used CCC to teach algebra to one 15-year-old boy with learning difficulties in a 5-week, single-subject intervention study, finding that CCC significantly improved the student’s fluency with simplifying algebraic expressions. However, the gains made by the student did not necessarily generalise to novel contexts, which raises questions about the wisdom of directly addressing mathematical fluency in isolation (Foster, 2018; Heinze, Star, J. R., Verschaffel, 2009; Joklitschke et al., 2022; Liljedahl, 2020).

These studies suggest that CCC may be feasible for secondary mathematics. However, there do not yet exist any peer-reviewed empirical studies investigating the effectiveness of CCC with secondary-aged mathematics students without cognitive or behavioural disabilities. This might be because CCC has mostly been used to teach arithmetic facts, rather than the kinds of multi-step procedures typically found in mainstream secondary education. It could also represent a wider trend in the computational, mathematical and procedural fluency literature for studies to focus on younger students. It would seem that many secondary-level content areas in mathematics involve procedural skills where fluency is important (Foster, 2018), and it seems worthwhile to examine whether CCC might be an appropriate way to develop such fluency.

Another possible reason for the absence of studies on CCC in secondary mathematics might be difficulty of implementation. Implementing CCC in a classroom setting can be cumbersome (Musti-Rao & Plati, 2015), and treatment fidelity of CCC has been observed to be low in some primary-school classrooms (Byron et al., 2021). While a CCC task to practise factual recall, such as multiplication tables or spellings, might be printed on a single sheet, and covering and comparing the model could be relatively straightforward, designing a paper-based CCC task for more complex mathematical procedures might be challenging. Here, a designer would need to create space for the questions, the model solution and the students’ responses — all of which might take up several lines — and possibly even space for the students to make corrections afterwards. Thus, not only might implementing a secondary-level CCC activity require a lot of preparation and instruction time, and be costly in terms of printing resources, but students would be required to manage this activity themselves. This means that they would be responsible for ensuring that they attempt each question in order and maintain fidelity to the CCC process, including covering the model and checking their solution against the model to identify potential mistakes. All of this seems potentially more difficult as procedures become more complex. Another barrier to the use of paper-based CCC in schools could be the investment of time needed for students to become familiar with the CCC process itself.

A software application (app) may have the potential to improve the fidelity of CCC and circumvent these practical problems when using CCC with longer procedures. An app organises the tasks for the students, automatically records their data and offers the potential for individualised learning through tutorials. Apps can also promote standardisation across trials and research contexts and sustain high participant fidelity (Al-Ubaydli et al., 2021). Additionally, using an app allows researchers to collect detailed data that can be used to examine the components that may underlie the CCC process (Konrad & Joseph, 2014; Riccomini et al., 2017).

### Research Questions

In summary, there is strong evidence for the effectiveness of CCC when it is used in primary mathematics to train computational fluency (Alptekin & Sönmez, 2022; Joseph et al., 2012). However, no study has yet designed and evaluated CCC tasks for mainstream secondary mathematics that seek to improve students’ procedural fluency on multi-step tasks. Given the potential limitations of paper-based CCC for secondary mathematics, in this study we design and evaluate the effectiveness of a novel CCC app, which we call *CCC:Graphs*. The content area chosen for this study is finding the equation of a straight line given two points. We chose this content because it is a commonly encountered topic in school mathematics at many age levels and is possible to address using a well-defined procedure that we do not anticipate varying too much between different schools.

In this paper, we describe a randomised controlled trial (RCT) in which we compared *CCC:Graphs* to a similar app designed by the first author, not based on CCC, intending to simulate, in the same technological environment, the sorts of exercises students might typically complete in a secondary mathematics lesson (from here referred to as ‘standard exercises’).

The research question for this study is the following: *How effective is CCC:Graphs, compared to standard mathematics exercises on an iPad, at developing students’ procedural fluency with finding the equation of a straight line given two points?*

## Method

All intervention resources, including the tests, lesson plans and videos, can be found at https://github.com/jda5/ccc-graphs-production. The source code of the apps and analysis can be found at https://github.com/jda5/ccc-graphs-rct-analysis and the pre-registration is at https://osf.io/f7zgd.

### Study Design

Ethical approved was granted by Loughborough University’s Ethics Approvals (Human Participants) Subcommittee and the study was pre-registered prior to data collection (https://osf.io/f7zgd). The study adopted a two-group, between-subjects experimental design, with one independent variable (group allocation: *CCC:Graphs* app or control app) and three dependent variables: pre-test, post-test and delayed post-test (1 week later). Students in participating classes were randomly allocated to either the experimental group (using the *CCC:Graphs* app) or the control group (using an app based on standard exercises). We implemented the study across one ordinary mathematics lesson, which is the amount of time typically allocated to this content area in the schools’ teaching timetables. This supports an authentic and realistic implementation that is not artificially increased by using a larger than typical period.

First, a baseline measure of the students’ procedural fluency was taken in a pre-test, focused on finding the equation of a straight line given two points on the line. Then, after using either the experimental app or the control app for about 35–40 min, students’ procedural fluency was measured again in a similar post-test. Finally, a week later, a third measure of students’ procedural fluency was taken in a similar, delayed post-test. As such, the dependent variables are the students’ scores on the post- and delayed post-test, the independent variable is students’ group allocation (*CCC:Graphs* or control), and the covariate is the students’ scores on the pre-test.

The hypotheses of this study are:

- H_1 post_: The post-test scores of the experimental and the control groups are different, after controlling for the pre-test scores.
- H_0 post_: There is no difference between the post-test scores of the experimental and the control groups, after controlling for the pre-test scores.
- H_1 delayed_: The *delayed* post-test scores of the experimental and the control groups are different, after controlling for the pre-test scores.
- H_0 delayed_: There is no difference between the *delayed* post-test scores of the experimental and the control groups, after controlling for the pre-test scores.

To ensure participants’ anonymity, each student was assigned a unique four-digit ID number. The ID number additionally encoded information about the students’ school and class, allowing the researchers to cross-reference students’ test responses and their app data.

Participants were not told which condition they had been assigned to. Both groups were told that what they were writing on the apps would be assessed to better understand how secondary school students learn how to find the equation of a straight line given two points. Participation was voluntary.

**Fig. 1:**
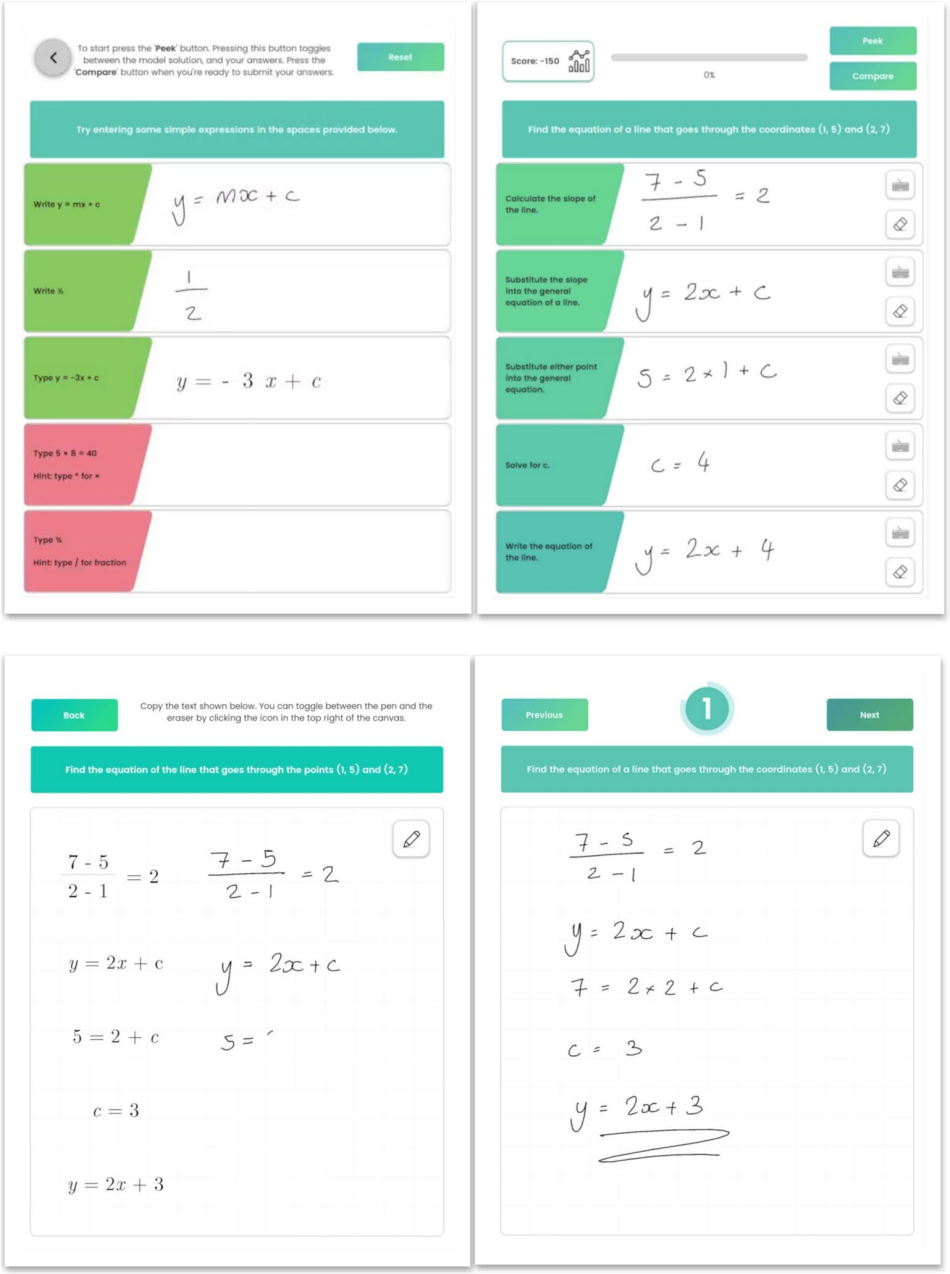
A side-by-side display of CCC:Graphs app (top) and control app (bottom). The left screen depicts the tutorials, and the right screen depicts the task, with partial annotations from students. The solution is purposefully incorrect to demonstrate how the app handles incorrect responses

Participants were given an iPad (Air, 3rd generation, running iOS 15) with the app pre-installed, a digital pen (Apple Pencil, 1 st generation) and a pair of headphones. They were instructed to follow a link to a 3-min video tutorial (the above link) that explained what CCC is and how they were expected to use the app. The students watched the video tutorial on their iPad, with their headphones plugged in, so that they could view it at their own pace without disturbing other participants. After watching the video, the participants were instructed to complete the tutorial activity (see Fig. 1) before starting the task. They completed the task at their own pace, with no support from either the researcher or the classroom teacher.

To obtain the best possible baseline measure of participants’ procedural fluency prior to the intervention, the pre-test was given immediately before starting the intervention. This gave the participants approximately 35–40 min during the intervention on the apps (as part of an hour-long mathematics lesson). The post-test was administered the following day, at the start of the participants’ regular mathematics lesson, and the delayed post-test was administered 1 week after the intervention, again at the start of a regular mathematics lesson. Participating students were not taught in class about linear graphs in the fortnight leading up to the intervention, nor was it taught in the week between the intervention and the post-test.

### Sampling and Participants

The participants eligible for this study were secondary school students in England, in years 9, 10 or 11 (aged 13–16). We sought students with some basic knowledge of linear graphs (i.e. of the general equation of a straight line in the form $y=mx+c$, where $m$ is the gradient and $c$ the *y*-intercept) but who were not expected to be able to confidently find the equation of a straight line given two points. Due to time constraints, we did not screen potential participants on their ability to find the equation of a straight line prior to including them in the study; instead, we relied on the teachers in the participating schools helping us identify suitable classes.

The headteachers of six mainstream, co-educational secondary schools in England were contacted via email and asked if the students attending their schools would be interested in participating in the study. We selected these six schools based on convenience, using contacts known to us. Five of the schools agreed, and each nominated two mathematics classes that the head of mathematics thought best met the eligibility criteria. Class sizes in this study ranged from 11 to 30 students. The median number of students on roll for the five schools was 980, with an interquartile range of 160.^3^ Three of the schools were situated in urban areas and two in rural areas. Of the five schools, three had a set of iPads available for use in the students’ regular lessons, which teachers said were used occasionally, but for this study we provided iPads with the apps pre-installed.

We obtained ethical approval for an opt-out recruitment protocol, meaning that opt-in parental consent was not required, since the activities that the students were given were under the control of their teacher and classed as normal classroom activity. Two weeks prior to the start of the intervention, parents of participating students were sent information about the study. They were given the opportunity to decline participation following the initial mailing and were then contacted a second time, 1 week before the start of the intervention, if they had not already opted out. On the day of the intervention, students were given a verbal and printed outline of the study and were told of their right to withdraw at any time, without needing to provide a reason. They were then given the opportunity to ask questions about the study. After this, students were asked to sign a form confirming their willingness to participate. Students opting out still used one of the two apps during their mathematics lesson, and sat the three tests, but any data collected from them were removed before analysis.

To determine the number of participants needed for this study, we conducted a power analysis using G*Power 3.1 (Faul et al., 2007). To increase the robustness of our study and, in particular, to mitigate against Type II errors, we planned for 95% power (rather than the more usual 80%) to detect a medium-sized *f* effect size of 0.25, with the standard 5% alpha significance level. Studies on CCC tend to be single-subject studies (Joseph et al., 2012), where the percentage of nonoverlapping data (PND) is a commonly used measure of effect size, and it is not possible to convert this into an effect size based on variance explained (e.g. ${\eta }^{2}$ or Pearson’s *r*). As such, we estimated that *CCC:Graphs* might produce a ‘medium’ effect of *f* = 0.25 (Cohen, 1988), based on Joseph et al.’s (2012) meta-analysis, which found that CCC is effective in students with and without special educational needs (72.4% PND — interventions are considered effective if the PND is between 70 and 100% [Scruggs & Mastropieri, 2001]).

We planned for a 2 × 2 between-subjects ANCOVA to determine whether there were differences between the post- and delayed post-test scores of the experimental and the control groups, with pre-test scores as a covariate. Using G*Power, this gave a total sample size of 210.

In total, 229 secondary school students in years 9, 10 and 11 (ages 13–16) across the five schools were invited to participate. Three students opted out and two students were dropped from the study due to non-compliance (the students did not engage with the intervention), resulting in a final sample of 224 that took the pre-test. Of these 224 students, 213 sat the post-test and 171 sat the delayed post-test. The sizable delayed post-test dropout was due to data loss: one of the schools lost the tests that they had given to their students and, despite their best efforts to locate them, were not able to return their papers. Since the data were anonymised, it is not possible to link data to particular schools. However, the random experimental design means that we can expect that variations between the schools should balance out between the control and experimental groups, and so we do not think that this loss of data should materially affect the conclusions of the study. A complete breakdown of the group sizes is shown in Table 1. The dropout rate for the post-test is 4.2% and 22.7% for the delayed post-test, due to this problem of test paper loss.

**Table 1:** The number of participants in each condition that took the pre-test, post-test and delayed post-test

	**Pre-test**	**Post-test**	**Delayed post-test**
Experimental	119	114	92
Control	105	99	79
Total	224	213	171

### Materials

In this section, we outline the development, features and theory that informed the design of the experimental and control apps, framed around Riccomini et al.’s (2017) three key elements of effective fluency practice: (i) modelling, (ii) multiple opportunities to respond, and (iii) immediate feedback.

#### CCC:Graphs App

*CCC:Graphs* is an app designed to be used on an iPad with an Apple Pencil. On starting the main task, the user begins the CCC activity. A task is displayed at the top of the screen: ‘Find the equation of the line that goes through the points $({x}_{1},{y}_{1})$ and $({x}_{2},{y}_{2})$.’, where the letters ${x}_{1},$ ${y}_{1}$, ${x}_{2}$ and ${y}_{2}$ are replaced with integers.

##### Modelling

The user is also shown a model solution, broken into five steps, along with their score and a button that reads ‘Ready’. Figure 1 shows both CCC:Graphs and the control app, with a tutorial screen and task screen for each, partially completed by students (including an incorrect response from one). The tutorial videos shown to the students (available at https://www.youtube.com/watch?v=EgN4KQdHITA and https://www.youtube.com/watch?v=qM_3imPGbqY) clarify further how the app looks and feels. The user can study the model solution for as long as they wish.

##### Multiple Opportunities to Respond

The student then clicks the ‘Ready’ button, and the model solution disappears and is replaced by empty fields, in which the user is expected to write their response to the same task, using the digital pen. The app also displays some text to the left of each step that is designed to prompt the user on what to do. These steps were (i) calculate the gradient of the line, (ii) substitute the gradient into the general equation of the line, (iii) substitute either point into the general equation, (iv) solve for $c$, and (v) write the equation of the straight line.

##### Immediate Feedback

The users were also shown a score in the top left corner. The scoring system was designed to gamify the task and encourage users to focus on submitting correct responses. Users are awarded 100 points for each correct step. If the user is unable to respond to the task, they are given the option to ‘peek’ at the model again, by pressing a button that reads ‘Peek’. Doing so brings up the model, and subtracts 150 points from the user’s score.

Allowing the user to peek at the model solution gives them the opportunity to self-manage their learning and to successfully complete the task — both of which are designed to improve their sense of self-efficacy and general mathematics achievement (Rivers, 2001; Thomas, 1980; Usher, 2009). However, giving users the opportunity to peek at the solution whenever they wish could conflict with the purpose of CCC of having them remember the model. Though the number and length of peeks at the model solution was not restricted, we intended that the points deduction for peeking would discourage users from doing so excessively.

During piloting, we found that the students typically only read the text for the first task, and when they got stuck, rather than for each task. We further observed that students generally tended to peek rather than read the text when they got stuck. We concluded from this that the reading demands of the app were not a concern.

After the user has written their entire solution, they press the ‘Compare’ button and the app interprets what they wrote and marks their work. Correct responses are shown in green, while incorrect responses are shown in red. Users are then given the option to compare each individual response with the model. When ready to progress, the user presses the ‘Next’ button and the next task is displayed, along with the model answer. There are a total of 20 tasks in *CCC:Graphs*, intended to be increasing in difficulty, although the difficulty was not measured empirically. A video tutorial displaying all the features of the experimental app can be viewed at https://www.youtube.com/watch?v=EgN4KQdHITA.

##### Piloting

Author 1 designed and developed *CCC:Graphs* over about 1 year. The app was developed incrementally (Sommerville, 2011), and each new version was trialled with different users for comments, and was improved based on their feedback. Over the course of four rounds of trialling and development, a total of 24 education experts (teachers and researchers) and 18 secondary school students (aged 12–14) tested and gave feedback on different versions of the app. Trialling involved observations of the users interacting with the app for approximately 10–15 min, followed by audio-recorded semi-structured interviews of about 15 min. Data collected from these trials revealed bugs and compatibility issues and generated a proof of concept. The source code for *CCC:Graphs* and all its iterations (the prototype, alpha, beta and production versions) can be found at https://github.com/jda5/ccc-graphs-prototype, https://github.com/jda5/ccc-graphs-alpha, and https://github.com/jda5/ccc-graphs-beta.

#### Control App

As we have seen, CCC is a method for learning mathematical procedures that employs modelling, multiple opportunities to respond and feedback. As such, to evaluate the effectiveness of CCC, we compared it with a method of learning mathematical procedures consisting of identical tasks, in the same technological environment, but *without* these three components of CCC. Participants were presented with a task, on an identical iPad, with as similar an aesthetic look as possible, followed by a space where they were asked to write their solution (Fig. 1). The same tasks were presented in the same order as in the experimental condition, but *without* modelling the task, *without* breaking up the task into separate steps, and *without* providing feedback on the students’ solutions.

Given the relatively short duration of this intervention (a single mathematics lesson), as explained above, it was essential to attempt to mitigate any effects on learning based on the novelty of the task or use of technology. In the context of human performance, Tulvig and Kroll (1995) hypothesised that the encoding of information into long-term memory is influenced by the novelty of the information. Since then, studies have found evidence supporting this hypothesis (Kormi-Nouri et al., 2005), and have extended the hypothesis to include not only the novelty of the information but the novelty of the events (Barceló et al., 2002). The perceived novelty of mobile apps has been shown to increase students’ motivation, leading to higher rates of learning (Jeno et al., 2019). As such, to mitigate any confounding caused by the novelty effect, it was important that the control group students were also given an app to work on, along with a digital pen and headphones.

Consequently, the first author developed a bespoke app for the control group that satisfied these requirements (the source code can be found at https://github.com/jda5/ccc-graphs-control). This app was designed to be used on an iPad, was of a similar appearance to *CCC:Graphs*, featured the same tasks and had users submit their answers by writing on the iPad using an Apple Pencil. The control app did not feature any modelled solutions, did not offer students multiple opportunities to respond by breaking the task into its constituent parts, and did not give any feedback on participants’ performance. Students were instead presented with one large blank space in which to write their response. A tutorial video displaying all of the control app’s features can be viewed at https://www.youtube.com/watch?v=qM_3imPGbqY.

As in the experimental group, participants in the control group were given an iPad with the app pre-installed, a digital pen and a pair of headphones. They were also instructed to follow a link to a video tutorial (the above link) that explained how their app works. The tutorial activity aimed to give students some familiarity with the layout and mechanics of the app; this was essentially a ‘practice screen’ where students could experiment with using the app, becoming acquainted with its flow and mechanics, without affecting their score. During this time, students were permitted to ask questions about how the app worked and this was one of the only times during the experiment where teachers were allowed to help the students, provided that it was exclusively about how to use the app or how to do CCC.

Following the tutorial, students completed the task at their own pace, with no support from either the researcher or classroom teacher.

### Measures

#### Pre-, Post- and Delayed Post-tests

To measure changes in participants’ procedural fluency, the first author designed three tests of equal length and structure. Each test contained 16 tasks, written in the form ‘Find the equation of the line that goes through the points $({x}_{1}, {y}_{1})$ and $({x}_{2}, {y}_{2})$’, with ${x}_{1}$, ${y}_{1}$, ${x}_{2}$ and ${y}_{2}$ replaced by integers (see Table 2). Participants were given 8 min to complete as many tasks as they could. For each task, they could be awarded 0, 1, 2 or 3 marks:

- 1 mark for correctly finding the gradient
- 1 mark for correctly finding the *y-*intercept
- 1 mark for correctly writing the equation of the straight line

**Table 2:** Four example tasks on the pre-, post- and delayed post-test, illustrating the parallelisation and ramping of the tasks

Second pair of coordinates greater	Second point easier to substitute	$m$	$c$	Pre-test	Post-test	Delayed post-test
No	No	$+$	$+$	$\left(2, 7\right) \text{and} \left(4, 9\right)$ $y=x+5$	$\left(2, 9\right) \text{and} \left(4, 11\right)$ $y=x+7$	$\left(2, 8\right) \text{and} \left(4, 10\right)$ $y=x+6$
No	No	$+$	$-$	$\left(1, 3\right) \text{and} \left(4, 15\right)$ $y=4x-1$	$\left(1, 4\right) \text{and} \left(4, 19\right)$ $y=5x-1$	$\left(1, 2\right) \text{and} \left(4, 14\right)$ $y=4x-2$
Yes	Yes	$+$	$+$	$\left(5, 27\right) \text{and} \left(2, 12\right)$ $y=5x+2$	$\left(5, 23\right) \text{and} \left(3, 15\right)$ $y=4x+3$	$\left(5, 26\right) \text{and} \left(2, 11\right)$ $y=5x+1$
Yes	Yes	$+$	$-$	$\left(5, 25\right) \text{and} \left(3, 13\right)$ $y=6x-5$	$\left(4, 26\right) \text{and} \left(2, 12\right)$ $y=7x-2$	$\left(6, 26\right) \text{and} \left(4, 14\right)$ $y=6x-10$

Hence, the maximum score a participant could obtain on each test was 48. We avoided including any graphs, such as horizontal lines, whose equations could be written down by inspection, without working, and we did not see any evidence of students being successful while omitting steps.

We attempted to formulate three parallel series of ramped tasks that were as similar as possible across the pre-, post- and delayed post-tests. We did this by systematically controlling features such as:

(i) Whether the second pair of coordinates were (both) larger than the first pair of coordinates, making calculation of the gradient easier
(ii) Whether the gradient or intercept were positive or negative
(iii) Whether we perceived it to be simpler to substitute the second pair of coordinates or the first pair of coordinates to find the intercept

These are illustrated in Table 2 for four example tasks. We did not statistically determine the relative difficulty of each test or the success of the intended ramping.

To simplify implementation across multiple research sites and minimise the need for additional resources, participants were not permitted to use calculators during the assessments. To accommodate this, test items were intentionally designed using only integer values and limited to single-digit multiplication or multiples of 10. Partial marks were not awarded in the case of arithmetic errors.

#### App Data

An important challenge in conducting RCTs is to look beyond the overall effectiveness of the intervention under investigation. Evaluating the potential impact of the context in which the research is situated, or analysing different subgroups within a sample in relation to an intervention’s effect, are just some of the ways in which a researcher could better understand why an intervention may have produced its observed outcomes (Connolly et al., 2018). Thus, in addition to the tests, *CCC:Graphs* also collected time-stamped logs of how the students interacted with different elements of the app. These data included the time spent on each screen; a timestamp of each button press (i.e. peek, compare, next, etc.); a timestamp of each submitted solution, whether the solution was marked as correct, for which steps the student compared their solution; and the ASCII converted text of the students’ submissions. These logs were written to a CSV file and saved on the tablet (all log files can be viewed and downloaded in the link provided in https://osf.io/f7zgd).

Furthermore, on start-up, *CCC:Graphs* asked users to submit a four-digit ID number, as explained above, so that their app data could be paired with their test scores. This data enabled us to examine how students used *CCC:Graphs* throughout the intervention and whether differences in students’ app usage were associated with differences in improvements in procedural fluency. It also allowed us to investigate class- and school-wide differences in app usage and fluency outcomes.

### Procedure

The study took place during three of the students’ mathematics lessons and lasted a total of 80 min. All schools in this study had 60-min mathematics lessons, but other tasks needed to be completed during these lessons, including the pre-test, participant information and consent and the post-test and delayed post-test. The regular classroom teacher was present throughout, but was asked *not* to help their students with any of the mathematical content of the apps. Helping students manage the equipment — such as logging onto the school’s Wi-Fi or reconnecting the Apple Pencil — was allowed, though rarely needed.

One school scheduled back-to-back (double) mathematics lessons for their students, and so the students completed the pre-test, intervention and post-test all on the same day, with a 15-min break (as scheduled by the school) immediately before the post-test. However, the other four schools completed the post-test on the following day. All of these schools gave the delayed post-test at the start of one of their mathematics lessons a week later.

Before the start of the first lesson, the first author generated a random sequence of integers ranging from 1 to the number of students in the class, using https://www.random.org/sequences/. Then, walking around the classroom, front to back and left to right, he placed iPads on the students’ desks, giving them the experimental app if the corresponding random number in list was odd and a control app if the number was even. He then distributed the headphones, pens, pre-test and assent forms. Once the students were seated, he introduced himself and the study, gave the students an opportunity to ask questions, and then asked the students to sign the assent form. The students completed the pre-test and then watched a 3-min video on finding the equation of a straight line given two points on the line.^4^ This video was created for this study, so that all students were given the requisite mathematical knowledge to complete the app tasks, and to ensure that students across schools were given identical explanations.

Students were then instructed to start the app on their iPad, plug in their headphones and watch the video tutorials from links in the app’s home screen. At the end of each video, both groups were instructed to complete the tutorial activity in the app. The video tutorial for the control app is 90 s shorter than the one for *CCC:Graphs*, and students in the control group spent on average 29 s less time on the tutorial ($M_\text{ctr}=3.98\;\text{min}$ compared with $M_\text{exp}=5.97\;\text{min}$; time includes the video). This is because the control app is simpler, with fewer features, fewer screens, etc., and so does not require as much training. This also means that participants in the control group spent approximately 2 min more on the main task than those in the *CCC:Graphs* group did.

Students spent on average 20 min on the task ($M=20.24$ min, $SD=4.95$ min) and all groups were asked to work in silence. The tasks were not presented in a randomised order, since they were ramped in intended difficulty. Since control and experimental groups were given the same tasks in the same order, any ordering effects should not affect our conclusions. We deliberately presented many more tasks than we believed that students would be able to complete in the time available, so as to prevent students from finishing and having nothing to do, and in order to avoid ceiling effects. Occasionally students would ask for help in completing the tasks, but no additional support was given. Those in the experimental group were reminded that they could ‘peek’ at the answer if they wished, whereas students in the control group were told to write down anything that they thought was relevant, or, failing this, what part of the task they were stuck with.

In total, we collected 119 app log files that detailed the students’ interactions with *CCC:Graphs* (one for each participant in the experimental group) and 18,209 time-stamped data points — on average, 153 per participants.

### Analysis

To test the hypotheses of this study, we conducted a repeated-measures 2 × 2 analysis of covariance (ANCOVA) with one between-subjects factor (group: control, experimental), one with-subjects factor (time: post-test, delayed post-test) and pre-test as covariate, to determine whether there was a statistically significant difference between the use of the *CCC:Graphs* and control app on the post- and delayed post-test scores after controlling for students’ pre-test scores. These analyses were conducted in *Python* and the analysis script and data are available at https://github.com/jda5/ccc-graphs-rct-analysis.

Analysis of the app log data was exploratory, and consisted of data visualisations and descriptive and inferential statistics. We used *Python* with *Jupyter Notebook*^5^ to analyse the log data. Our analysis was divided into four phases. First, the data was processed so that key variables could be analysed at both the individual and sample levels. To achieve this, we used Python’s object-oriented paradigm to create *Participant* and *Question* objects that stored and modified the test data, and app data pertaining to the modelling and feedback components of *CCC:Graphs*. After this, we identified outliers and cleaned the data by creating figures and inspecting for outliers. In the third phase, we sought to gain an overview of the data over time, observe potential relationships among variables, examine clusters of participants and highlight promising areas for further analysis. During this phase, we primarily examined trends by creating figures and examining correlations. We also considered different approaches to clustering participants by experimenting with different clustering algorithms and variables on which to form clusters. Finally, in the fourth phase we further explored the areas of interest. We examined the differences between two clusters of participants attaining similar pre-test scores but dissimilar post-test scores, and investigated differences in fluency outcomes at the classroom and school level.

Analysis of the app data was exploratory and, given the number of analyses conducted, we do not report every one in this paper. However, readers can view and download the full Notebooks and the app data at https://github.com/jda5/ccc-graphs-app-data-analysis.

## Results

### Test Data

The mean and standard deviation of the pre-test, post-test and delayed post-test scores for both the experimental and the control groups are shown in Table 3. The data satisfied the ANCOVA assumptions of homogeneity of variance, homogeneity of regression slopes and linearity, except that the distribution of the post-test and delayed post-test scores departed significantly from normality, and there was a floor effect, indicating that the tasks were experienced as difficult. However, given a large enough sample size (i.e. > 40), violations of normality do not cause considerable problems (Ghasemi & Zahediasl, 2012), and empirical significance levels calculated from an ANCOVA are relatively unaffected by nonnormal distributions when there are no major differences in group size and there is homoscedasticity (Levy, 1980). Hence, we proceeded with the ANCOVA, but for completeness we also conducted a Kruskal–Wallis test on the difference in pre- and post-test outcomes, to obtain a non-parametric measure of the differences between the conditions on the dependent variables.

**Table 3:** The mean and standard deviation (in brackets) of the test scores for the experimental group and control group

	**Pre-test**	**Post-test**	**Delayed post-test**
Experimental	5.05 (9.63)	18.9 (13.3)	21.6 (13.9)
Control	5.01 (9.33)	16.0 (12.5)	17.8 (14.7)

The repeated-measures ANCOVA revealed a statistically significant main effect for group, $F\left(1, 167\right)=7.38$, $p=.007$, ${{\eta }_{p}}^{2}=.042$. There were no statistically significant effects for time (Wilks’ lambda $=.985$, $F(1, 167)=.265$, $p=.112$, ${{\eta }_{p}}^{2}=.015$) or the interaction between time and group (Wilks’ lambda $=.998$, $F(1, 167)=.265$, $p=.608$, ${{\eta }_{p}}^{2}=.002$).

The Kruskal–Wallis test also found a significant difference between the gain (post-test minus pre-test scores) of the experimental group ($Mdn=12.5$) and the control group ($\textit{Mdn}=10.0$), $H=4.65$, $p=.031$, ${\eta }^{2}=.017$. There were also significant differences between the *delayed* gain (*delayed* post-test minus pre-test scores) of the experimental group ($\textit{Mdn}=14.0$) and control group ($Mdn=10.0$), $H=4.65$, $p=.031$, ${\eta }^{2}=.026$. All of these tests indicate that the experimental group improved more than the control group at both the post-test point and the delayed post-test point.

### App Data

Analysis of the app data showed that most students in the experimental group completed the first nine tasks, spending on average 2 min and 28 s per task on the app, although this varied greatly between participants. Overall, students correctly submitted a median of four out of five steps per task, with step 3 — in which they needed to substitute either of the two given points into the general equation of the line — scoring the lowest percentage of correct responses. Since, as mentioned above, we deliberately avoided equations that we anticipated could be spotted by inspection, in order to get the last step correct, the student would need to get all of the previous steps correct and correctly determine both the gradient and the intercept. Given that the last step is contingent on the previous ones, the final step is very likely to be the one with the fewest correct responses.

We also observed a negative trend in the average time per task over the first nine tasks — a measure that is the sum of time spent viewing the model, answering the task, peeking and comparing solutions. Participants typically spent the most time on the first task, presumably because they needed to familiarise themselves with the app and the procedure. Times decreased overall by approximately 28% to a new normal for tasks 2 and 3. They then rose slightly for tasks 4 and 5, before spiking at task 6 and then returning to a plateau for tasks 7 onwards.

Participants studied the model solution for an average of 20 s before attempting the task themselves. Our analysis also showed that about half of the students (46%) sought additional feedback from the app on submitting an incorrect solution, spending on average 8 s comparing their solution with the model.

Most participants did not use the peek feature; there was a median of 0 peeks per task. The mean percentage of participants peeking four or more times per task was 9.4%, suggesting that some of those participants may have used the peek function to copy the entire model. Only 46% on average compared their solution with the model after submitting at least one incorrect step, and 33% of participants never compared their solution with the model after submitting fewer than five correct steps.

**Fig. 2:**
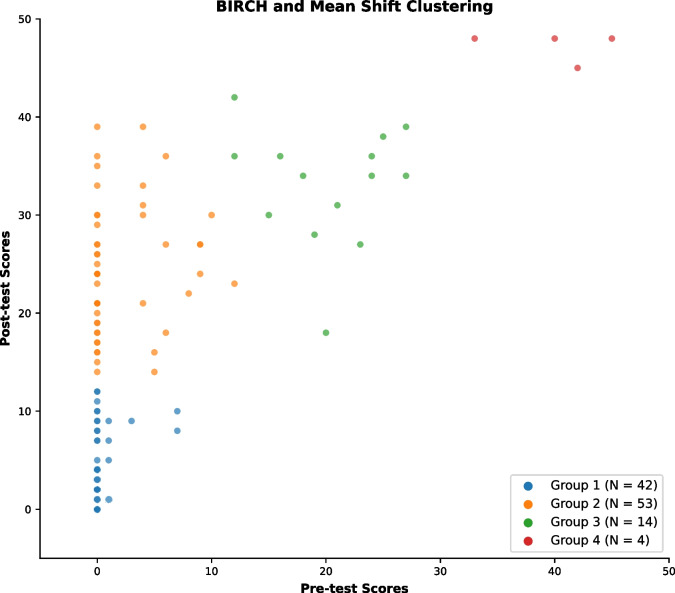
A scatter graph plotting participants’ pre-test scores against their post-test scores and their group as a result of BIRCH and mean shift clustering

To better understand the differences in app usage between students who made low and high fluency gains, we clustered participants based on their pre-test and post-test scores using the mean shift (Cheng, 1995) and balanced iterative reducing and clustering using hierarchies (BIRCH) (Zhang et al., 1996) clustering algorithms. These algorithms produced identical results. For mean shift, we set the bin seeding parameter to *True* and changed the quantile parameter of the bandwidth estimator to 0.2, as these values resulted in a good number of clusters. The number of clusters was determined by discerning an ‘elbow’ in a plot of distortion against the number of clusters (Cheam & Fredette, 2020). Mean shift produced four clusters. For BIRCH, we adjusted the number of clusters from 3 to 4 and used the default values that *scikit-learn*^6^ implements for all other parameters. Setting the number of clusters to four, BIRCH gave the same clustering as mean shift (see Fig. 2).

Of particular interest were differences in the app usage between participants in groups 1 and 2 (as shown in Fig. 2). Both sets of participants achieved comparable pre-test scores, but group 2 achieved higher post-test scores. Consequently, we examined differences in app usage between these two groups to reveal if certain features of *CCC:Graphs*, or behavioural patterns in the students, correlated with their improvements in procedural fluency.

There were differences between the group 1 and group 2 clusters in the compare rates, with participants in group 2 comparing about 9% more than those in group 1 did. Participants in group 2 also completed more tasks, with 79% completing tasks 1 to 8, compared with only 59% in group 1. However, group 1 typically exhibited higher means for variables pertaining to peeking and viewing the model before attempting the task.

The key difference between the students in the two groups was the classroom that they were in. Group 2 consisted mostly of students (76.2%) from high-scoring mathematics classes for both experimental and control groups, while group 1 was mostly made up of students (77.4%) from low-scoring mathematics classes. We conducted a Kruskal–Wallis test and found that there were significant differences between the participating mathematics classes in the differences between pre- and post-test scores, $H\left(9\right)= 48.7, p<.001$. As a result, group membership was a rough indicator of class membership.

## Discussion

Both ANCOVA (${{\eta }_{p}}^{2}=.042$) and Kruskal–Wallis (${\eta }^{2}=.026)$ tests found significant differences in the post-test and delayed post-test scores, while accounting for the pre-test scores, between secondary school students using *CCC:Graphs* and those completing standard mathematics tasks on an iPad.

Our hypotheses were:

- H_1 post_: The post-test scores of the experimental and the control groups are different, after controlling for the pre-test scores.
- H_0 post_: There is no difference between the post-test scores of the experimental and the control groups, after controlling for the pre-test scores.
- H_1 delayed_: The *delayed* post-test scores of the experimental and the control groups are different, after controlling for the pre-test scores.
- H_0 delayed_: There is no difference between the *delayed* post-test scores of the experimental and the control groups, after controlling for the pre-test scores.

Our results support rejecting both null hypotheses, H_0 post_ and H_0 delayed_. We conclude that the experimental group outperformed the control group on both post-test and delayed post-test, controlling for pre-test scores. These findings provide evidence that *CCC:Graphs* is more effective at improving secondary school students’ procedural fluency with finding the equation of a straight line given two points than standard exercises implemented on an iPad.

It may be thought that neither group performed particularly well on the tests, since a score of less than 20 out of a possible 48 seems very low. However, as we explained earlier, we deliberately designed the tests so that it would be very hard for students to complete all of the tasks in the time permitted. This was more acceptable to the schools, given that students would be doing these tasks during lesson times, and faster students should not run out of tasks to do. Given that there was a minimum and a maximum score for each task, we wanted to avoid floor and ceiling effects. We attempted to mitigate floor effects by awarding students ‘method’ marks for deriving the gradient of the line and for identifying the intercept, hence increasing the sensitivity of the test. We also tried to minimise the risk of ceiling effects by designing the tests to be difficult to complete in their allotted time. With only 8 min to complete the tests, participants were allotted only an average of 30 s per task, which deliberately was considerably less than we expected to be necessary.

The present study adds to the very few studies that have explored the use of CCC in secondary school (age 11–18) in any subject (e.g. Althobaiti, 2020; Inouye, 2018; Morano & Aigotti, 2022) by providing evidence from an experimental trial in which CCC was implemented as an app to improve secondary school students’ procedural fluency. This study builds on Morano and Aigotti’s (2022) proposition that CCC could be used to develop fluency with procedural skills, and provides evidence that CCC can be effective in a mainstream secondary mathematics classroom. We acknowledge again, however, that procedural fluency can only ever be *part* of a student’s experience of mathematics, and we view fluency very much as a means to an end (i.e. reasoning and problem solving), rather than an end in itself (Liljedahl, 2020; Foster, 2023).

Analysis of the app data indicated that students in our study primarily focused on the modelling stage of CCC, and about half of students accessed the feedback elements of the app. The design of the app was based on the components of CCC outlined by Riccomini et al., (2017). As such, findings from the app data and the RCT offer some indirect support for the efficacy of secondary-level pedagogical approaches that incorporate modelling and feedback components. However, the app data alone was insufficient to assess the efficacy of any single one of these elements, and future research should investigate the relative contribution of these components to the development of procedural fluency.

We also identified a clear association between mathematics class membership and procedural fluency gains made using *CCC:Graphs*. This suggests that classroom-level factors (which may be a proxy for prior attainment) may influence procedural fluency gains made using CCC at the secondary level. Among the most commonly cited factors known to explain variance in mathematical outcomes between classes are attainment setting and socioeconomic status (Broer et al., 2019; Lamb & Fullarton, 2002). These factors may have influenced improvements in procedural fluency made using *CCC:Graphs*, but other factors such as the recency and extent to which the class had previously addressed the topic of linear graphs may also have been important.

To our knowledge, this study is also the first to implement CCC as an app, and demonstrates that an app based on CCC can be employed class-wide to manage the stages of covering a model, copying it and subsequently comparing it with students’ solutions. The use of an app also allowed participants in this study to individually watch videos explaining CCC and complete tutorials to help familiarise them with the process. Participants in the experimental group took just under 6 min on average to become familiar with CCC and, with the exception of two students whose app crashed, required no additional support while completing the task. This is particularly noteworthy, as teachers have previously perceived interventions based on CCC as time consuming and cumbersome (Musti-Rao & Plati, 2015).

### Limitations

This study controlled for the quality of instruction by showing all students the same instructional video and by denying students any individual help with the mathematical content of the app. We did not control for instruction prior to the intervention, nor for any support students might have received outside of lessons between the pre-, post- and delayed post-tests.

To mitigate any spillover effects between the control and experimental groups, the students completed the task in silence. However, this was not fully controlled for, as the two groups were not physically separated, and completed the tasks side-by-side. Having both groups complete the tasks in this way may have introduced some bias through reactive behaviours by those in the control group. Seeing their classmates working on a much more dynamic app may have made students aware of their status as members of the control group, resulting either in a feeling of demotivation or possibly even a drive to overcome the perceived disadvantage (Irving & Holden, 2013). Neither group was told of their assignment to either the control or experimental condition, and at the start of the intervention the students were told that the researchers of this study were interested in their answers to the tasks presented on the app.

The CCC app developed by the first author integrated features intended to assist the CCC process. These features may improve the user interface and experience, but they may also act as confounding variables or limiting factors. For instance, the app automatically notifies the student if the steps they submitted are correct or incorrect, whereas, in a traditional implementation of CCC, students are expected to establish the correctness of their responses themselves. It is unknown whether the process of determining the similarity or dissimilarity between a response and the model strengthens the students’ comprehension of the procedural skill. As such, this feature may have limited the overall effectiveness of CCC.

More controversial, however, is the ‘peek’ function. For one-step computational fluency, the notion of ‘peeking’ at the model (i.e. the student looking back at the model solution before completing theirs) is counter to traditional CCC, and has in other studies been considered as a form of treatment infidelity (Grafman & Cates, 2010). However, during the development of *CCC:Graphs,* it seemed necessary to allow the user to peek at the model in order to reduce the cognitive load of the multi-step task and prevent disengagement of users when they were unsure of a particular step. This design choice was then tested, and its utility verified, during the piloting phase of the app’s development. Nonetheless, peeking is unconventional for CCC, and claims made about CCC in secondary education based on the findings of this study should note that *CCC:Graphs* implemented some features additional to CCC, such as peeking, automatic response checking and the ability for students to view their performance data.

We also note that the focus of the app was on *functions*, thereby excluding vertical lines ($x=\text{constant}$). We also avoided including horizontal lines ($y=\text{constant}$) and other ‘obvious’ patterns of pairs of points, such as (3, 3) and (5, 5) for $y=x$.

Finally, we did not statistically determine the relative difficulty of each test or measure their validity or reliability, which may limit the precision of our findings and introduce bias when interpreting pre- and post-test results. Because the tests consisted of well-established, straightforward procedural questions, we considered face validity adequate for this exploratory work. Nevertheless, full formal validation would be a valuable focus for future work.

## Conclusion

The results of this randomised controlled trial suggest that the use of a CCC app is more effective than an app employing standard exercises in improving secondary school students’ procedural fluency with finding the equation of a straight line given two points on the line. Furthermore, this study provides evidence for the efficacy of technology-based interventions that incorporate modelling and feedback components. Procedural fluency is only one aspect of learning mathematics, and devoting lesson time to developing fluency must be justified by wider aims around supporting students’ sense-making and problem solving (Foster, 2018; Liljedahl, 2020). Nevertheless, this study establishes a warrant for the use of CCC in secondary mathematics education, albeit alongside complementary activities that promote student reflection, deeper conceptual understanding and flexible application of learned procedures. Further research is needed to investigate whether these findings generalise to topics beyond linear graphs. Additionally, research should examine the efficacy of CCC with secondary school students of other ages.

We also suggest that CCC might also be effective in post-secondary mathematics education. For instance, CCC could potentially be used to practise procedural skills in advanced mathematics, such as the chain rule used to find the derivative of composite functions or the solution of sets of simultaneous equations by Gaussian elimination. Furthermore, CCC does not strictly have to be a worksheet or an app; for instance, a teacher could model a procedure on the board, cover or remove it, and then ask students to try to reproduce it from memory. Research investigating different implementations of CCC could establish the generalisability of the findings of this study.

Finally, the data in this study alone was insufficient to assess the individual efficacy of modelling, opportunity to respond and feedback components, or to determine how variations in instructional approaches, such as step-by-step versus correct/wrong feedback, might influence outcomes. Future studies are required to better understand the relative contributions of, and interactions between, these three components to the development of students’ procedural fluency.

## Data Availability

The data generated and analysed during the current study are available on the linked GitHub pages (https://github.com/jda5/ccc-graphs-rct-analysis) and OS repository (https://osf.io/f7zgd). The data includes pre-test, post-test and delayed post-test scores, and app usage data.

## Footnotes

1 Please note that we are *not* here using the term ‘modelling’ in the sense of ‘mathematical modelling’.

2 *Explicit Timing* is a strategy aimed at improving students’ mathematical fluency by timing mathematical exercises in 30-min intervals. This approach involves assessment, timing and re-assessment of students’ fluency, and is intended to increase speed and accuracy in problem solving.

3 The median number of students on roll and the interquartile range have both been rounded to the nearest 10 to protect the anonymity of the participating schools.

4 This video can be seen here: https://github.com/jda5/ccc-graphs-production

5 *Jupyter Notebook* is a web-based interactive development environment that contains both code and rich text elements (mark-up). More information can be found at https://jupyter.org

6 *Scikit-learn* is a machine learning library for the Python programming language. Further information about the library can be found at https://scikit-learn.org/.
